# Effect of immunostimulation with bacterial lysate on the clinical course of allergic rhinitis and the level of γδT, iNKT and cytotoxic T cells in children sensitized to grass pollen allergens: A randomized controlled trial

**DOI:** 10.3389/fimmu.2023.1073788

**Published:** 2023-01-17

**Authors:** Kamil Janeczek, Wioleta Kowalska, Michał Zarobkiewicz, Dorota Suszczyk, Marek Mikołajczyk, Ewa Markut-Miotła, Izabela Morawska-Michalska, Adrian Bakiera, Aleksandra Tomczak, Agnieszka Kaczyńska, Andrzej Emeryk, Jacek Roliński, Krystyna Piotrowska-Weryszko

**Affiliations:** ^1^ Department of Pulmonary Diseases and Children Rheumatology, Medical University of Lublin, Lublin, Poland; ^2^ Department of Clinical Immunology, Medical University of Lublin, Lublin, Poland; ^3^ Independent Laboratory of Cancer Diagnostics and Immunology, Medical University of Lublin, Lublin, Poland; ^4^ Department of Allergology, Voivodeship Rehabilitation Hospital for Children in Ameryka, Olsztynek, Poland; ^5^ Department of Botany and Plant Physiology, University of Life Sciences in Lublin, Lublin, Poland

**Keywords:** allergic rhinitis, bacterial lysate, children, γδT cells, iNKT cells, cytotoxic T cells

## Abstract

**Background:**

There are many drugs for allergic rhinitis (AR), however, these drugs show variable clinical effectiveness and some side effects. Therefore, new methods of AR pharmacotherapy are being sought.

**Objectives:**

The objectives of this study were to evaluate the efficacy of polyvalent mechanical bacterial lysate (PMBL) therapy in improving the clinical course of grass pollen-induced AR (seasonal AR, SAR) in children and its effect on changes in the blood level of the γδT, iNKT and cytotoxic T cell subsets.

**Methods:**

Fifty children with SAR were enrolled in this study and were randomly assigned to either the PMBL group or the placebo group. The severity of SAR symptoms was assessed using the total nasal symptom score (TNSS) and visual analogue scale (VAS). During two visits (V1, V2), peak nasal inspiratory flow (PNIF) was measured and peripheral blood was collected for immunological analyses. The study also included 2 telephone contacts (TC1, TC2).

**Results:**

The severity of the nasal symptoms of SAR on the TNSS scale was revealed to have a significantly lower impact in the PMBL group vs the placebo group at measuring points TC1 and V2 (p = 0.01, p = 0.009, respectively). A statistically significantly lower mean severity of nasal symptoms of SAR on the VAS scale was recorded for children in the PMBL group compared to the placebo group at measuring points TC1, V2 and TC2 (p = 0.04, p = 0.04, p = 0.03, respectively). The compared groups do not show significant differences in terms of PNIF values at individual measuring points. There were no statistically significant changes in immune variables. For both groups, there was a statistically significant association between the level of Th1-like γδT cells and the severity of SAR symptoms expressed on the TNSS scale (p = 0.03) – the lower the level of Th1-like γδT cells, the higher the TNSS value.

**Conclusion:**

Administration of sublingual PMBL tablets during the grass pollen season proves to have a high efficacy in alleviating SAR symptoms in children sensitized to grass pollen allergens. Th1-like γδT cells may be used as potential markers for SAR severity in children.

**Clinical trial registration:**

ClinicalTrials.gov, identifier (NCT04802616).

## Introduction

Allergic rhinitis (AR) is one of the most common allergic inflammatory diseases worldwide and it has a significant economic impact through its effects on education, productivity and use of healthcare resources (1, 2). Importantly, it is also a significant risk factor for sinus infections, otitis, and the development or exacerbation of asthma (3).

There are many drugs for AR, including: intranasal corticosteroids, oral and intranasal H1-antihistamines, leukotriene receptor antagonists, intranasal anticholinergic, intranasal and oral decongestants, intranasal saline, allergen immunotherapy and anti-IgE therapy (4, 5). However, these drugs show variable clinical effectiveness, and some side effects or poor efficacy (1, 6, 7). Therefore, new methods of AR pharmacotherapy are sought (8, 9). Recent data indicate possible clinical benefits from the use of probiotics (10), phosphodiesterase 4 inhibitors (11), various methods of immunomodulation (12–15), biologic agents such as mepolizumab or dupilumab (16) and autologous gold-activated patient serum (17). Clinical trials from recent years indicate that bacterial lysates (BLs) may also be one of the options. These preparations are a mixture of bacterial antigens obtained by mechanical (polyvalent mechanical BL, PMBL) or chemical (polyvalent chemical BL, PCBL) lysis of the bacteria most frequently responsible for respiratory infections. It is postulated that the former, due to the lower degree of antigen damage, may have greater immunoregulatory properties (18). BLs have been successfully used in the treatment of recurrent respiratory tract infections for years (19). They have been shown to be highly effective in the treatment of both seasonal (SAR) (20) and perennial (PAR) (21–23) AR. They are widely available, relatively cheap, easy to take by patients, and well tolerated (24, 25).

γδT cells are a small subset of human T cells, comprising approximately 1–10% of T cells. They have a T cell receptor (TCR) built of γ and δ chains instead of α and β. These cells combine characteristics of both innate and acquired immune response and are frequently recognized as the ‘bridge’ between them (26). γδT cells are able to quickly produce and release significant amounts of pro- and anti-inflammatory cytokines and chemokines after being stimulated by several factors, including autoantigens, lipopeptides, and microbial antigens (27). As such, they play an essential role in human anti-infectious and anti-tumor defense. However, they also may contribute to autoimmunity and allergy (28, 29). It is postulated that γδT lymphocytes are involved in the development and modulation of the course of allergic diseases, however, this ambiguous role has not been yet confirmed (30, 31).

Another lymphocyte population capable of a sudden release of large amounts of cytokines is invariant natural killer T (iNKT) cells, a rare subpopulation (up to 0.5% of T cells) that combines features of typical T and NK cells (32). Activated iNKT cells secrete large amounts of IFN-γ and IL-4 as well as IL-2, IL-5, IL-6, IL-10, IL-13, IL-17, IL-21, TNF-α, TGF-β, and GM-CSF (33). Rapid synthesis of cytokines makes these cells important regulators of inflammatory processes, including allergic ones. It is postulated that these cells play a double role in the modulation of the course of allergy and its pathogenesis (34).

The purpose of this study was to evaluate the effect of PMBL therapy on the clinical course of grass pollen-induced AR in children, and primarily to characterize the immunomodulatory effects of this treatment.

## Methods

### Study design

It was a 1:1 randomized, double-blind, placebo-controlled study in parallel groups (PMBL vs placebo). The study received a favorable opinion from the Bioethics Committee of the Medical University of Lublin (Resolution No KE-0254/251/2020, 26 November 2020) and was prospectively registered with ClinicalTrials.gov (Trial Registration No NCT04802616, 17 March 2021). The study was conducted in accordance with Good Clinical Practice standards, and the ethical principles that have their origin in the Declaration of Helsinki. The project was implemented in 3 centers in eastern Poland from 22 March 2021 to 29 October 2021.

The primary objectives of this study were to evaluate the effectiveness of 3-month PMBL therapy in improving the clinical course of grass pollen-induced AR in children, and above all its effect on changes in the blood level of the γδT cell subsets: Th1-like, Th2-like, Th10-like, Th17-like, Treg-like; iNKT cell subsets: iNKT1, iNKT2, iNKT10, iNKT17, iNKTreg; cytotoxic T (Tc) cell subsets: Tc1, Tc2, Tc10, Tc17, Treg-like and to assess the relationship between the level of these lymphocytes and the severity of SAR symptoms assessed using the total nasal symptom score (TNSS).

The secondary study objectives were to assess the effect of PMBL therapy on the need for oral H1-antihistamines and intranasal corticosteroids during the grass pollen season and to evaluate the safety and tolerability of this therapy.

### Patients

Eligible for the study were children aged 5 to 17 years with SAR diagnosed, as per current ARIA recommendations (4), with predominant sensitization to grass pollen allergens determined by skin prick testing (wheal diameter ≥ 3 mm and greater than negative control; Allergopharma-Nexter Ltd, Poland) or based on serum allergen-specific immunoglobulin E (asIgE) levels (≥ class 2; Polycheck, Biocheck GmbH, Germany), with SAR symptoms present in at least two previous grass pollen seasons. Exclusion criteria were as follows: allergy to tree pollens, receipt of PMBL within 12 months or PCBL within 6 months before the randomization visit, oral or subcutaneous allergen-specific immunotherapy within 3 years before the start of the study, treatment with systemic corticosteroids within the last 6 months before the start of the study, history of respiratory infection within 2 weeks before the randomization visit, systemic immunologic disorders, history of transfusion of blood, blood components or blood products.

Children were recruited for the study in the second half of March 2021, before the start of the grass pollen season in Poland. For all patients, written informed consent was obtained from a parent or legal guardian and assent was obtained from the patient.

### Interventions

The study used PMBL obtained by sonification, whereby ultrasound is used to inactivate the bacterial cell wall (Ismigen, Lallemand Pharma AG, Switzerland). The product is available in the form of sublingual tablets containing 7 mg of bacterial lysate of the following bacteria: Streptococcus pneumoniae, Streptococcus pyogenes, Streptococcus viridans, Staphylococcus aureus, Neisseria catarrhalis, Haemophilus influenzae, Klebsiella pneumoniae and Klebsiella ozaenae. PMBL was taken by patients according to its dosage regimen in recurrent respiratory infections. Thus, children in the PMBL group received 3-cycles of PMBL treatment, each consisting of sublingual administration of one PMBL tablet on an empty stomach for 10 consecutive days followed by a 20-day break (25). In the control group, the patients received placebo tablets indistinguishable from the PMBL tablets in shape, color, smell/taste and dissolution time under the tongue.

### Randomization and masking

The randomization list was generated by hospital pharmacy staff using Random Allocation Software version 2.0. Pharmacists also prepared identical packs containing 30 PMBL or placebo tablets each and labelled them with a unique code consistent with the randomization list. The individuals responsible for preparing the randomization list and tablet packs were not involved in the implementation of the study. Children attending the first visit and meeting all inclusion criteria and none of the exclusion criteria drew a card with a unique package number. Patients and investigators, including those responsible for immunoassays, were blind to allocation. Unblinding was performed after receiving patient diaries from all participants, that was in early November 2021.

### Measurement of grass pollen concentration in ambient air

The dates of visits to the centers and the periods of assessment of SAR symptoms subjected to statistical analysis were determined based on measurements of grass pollen grain concentrations in ambient air.

**Figure 1:**
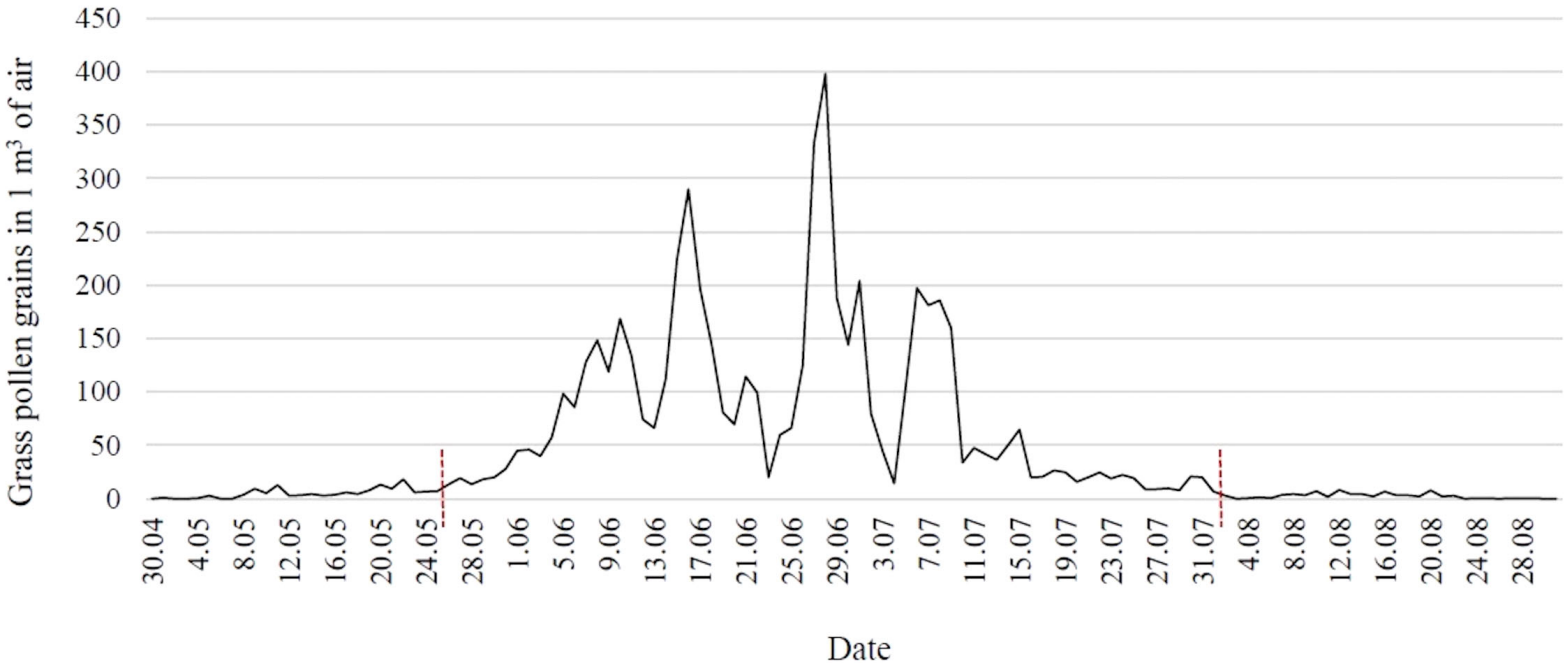
Concentration of grass pollen grains in the air in eastern Poland in 2021 (red lines indicate the time frame of the pollen season).

A Hirst-type apparatus (Lanzoni VPPS 2000) (35) placed on the flat roof of the building of the University of Life Sciences in Lublin (22032’25’’ E and 51014’37’’ N; 197 m a.s.l.) at 18 m above ground level was used to monitor the concentration of grass pollen grains in the air. The apparatus operated in continuous mode, sucking in air along with bioaerosol elements. The pollen grains stuck to a sticky-coated tape placed inside the apparatus on a moving drum. After a week’s exposure, the tape was cut into sections corresponding to 24 hours and microscope slides were prepared. The results were expressed as the number of pollen grains in 1 m^3^ of air per day. Pollen monitoring was conducted in accordance with the recommendations of the International Association for Aerobiology and Quality Control Working Group (36). The beginning and end of the grass pollen season in eastern Poland were determined using the 95% method (**Figure 1**) (37).

### Study protocols

**Figure 2:**
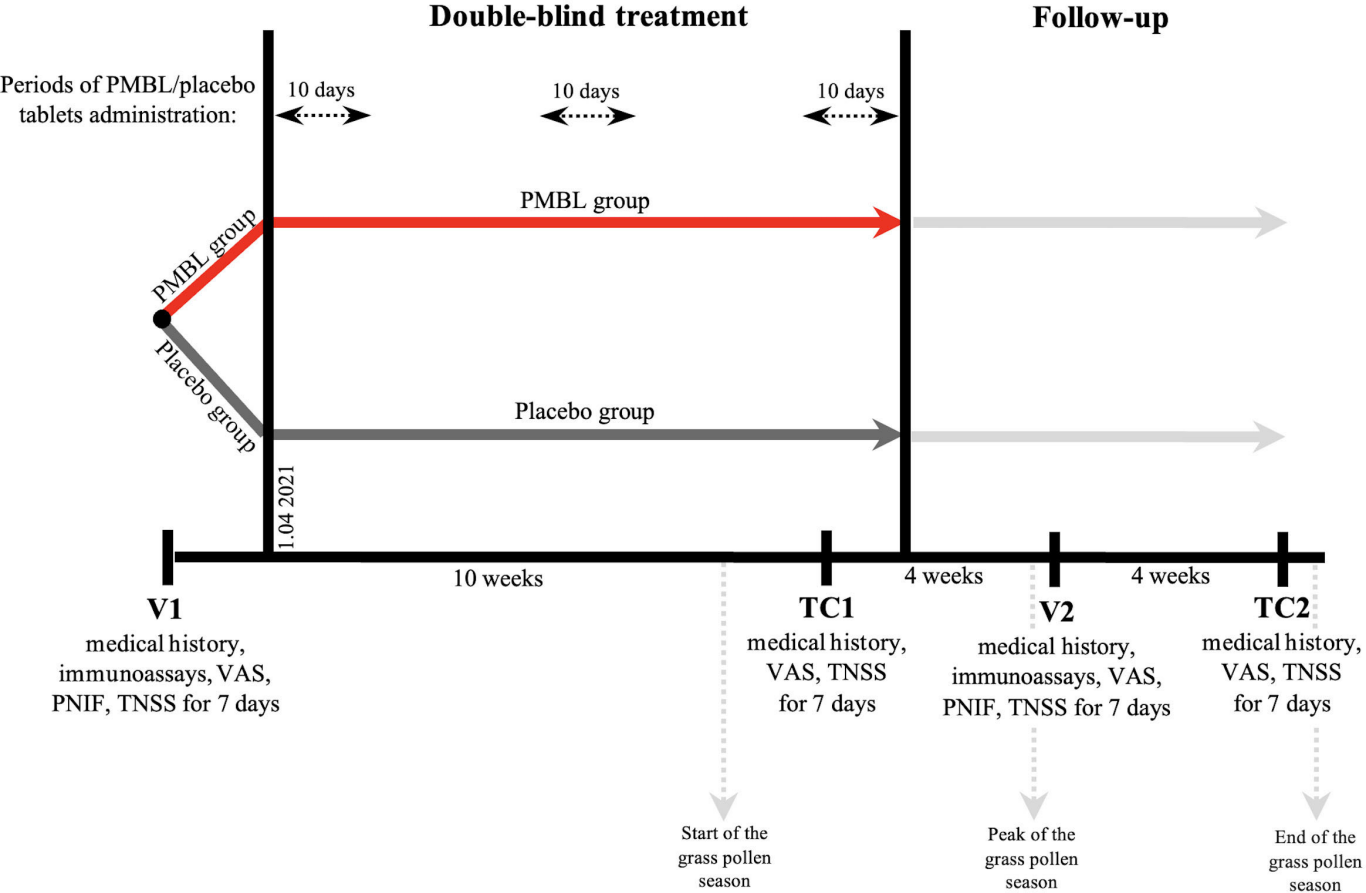
Study design. PMBL, polyvalent mechanical bacterial lysate; VAS, visual analogue scale; PNIF, peak nasal inspiratory flow; TNSS, total nasal symptom score.

The study included two site visits: visit 1 (V1) – a screening/randomization visit, before the start of the grass pollen season (22–31 March 2021), visit 2 (V2) – at the peak of the grass pollen season (23 June – 2 July 2021), and two telephone contacts (TC1, TC2; the study protocol registered at ClinicalTrials.gov provided for 4 telephone contacts, but two contacts were dropped due to the late start of the grass pollen season) (**Figure 2**). For 7 days after each visit, parents rated the severity of four SAR symptoms (nasal congestion, rhinorrhea, itching of the nose and sneezing) in the patient’s diary, each scoring from 0 to 3 (0 – no symptom, 3 – severe symptom), and then summed the scores to obtain the TNSS (weekly averages were analyzed statistically) (38). Patients started the first cycle of sublingual tablets on 1 April 2021. In addition, the use of an oral antihistamine (desloratadine) or an intranasal corticosteroid (mometasone furoate) was allowed in case of severe allergy symptoms, and the intake of these drugs was recorded daily in the diary (the number of days of the grass pollen season in which patients took the above drugs was analyzed statistically).

During the visits to the center (V1, V2), blood was taken for immunological tests, the severity of four SAR symptoms was assessed cumulatively on a visual analogue scale (VAS), and peak nasal inspiratory flow (PNIF) was measured using the Youlten Peak Flow Meter (Clement Clarke International, UK) (all subjects received appropriate instructions on how to use the Peak Flow Meter correctly; three measurements were recorded for each subject and the highest flow was used for analysis) (39). During telephone contacts, patients were asked to rate the severity of their SAR symptoms cumulatively on a VAS scale using an appropriately prepared tool in a patient diary (instructions, 10 cm line).

### Immunological analyses

The peripheral blood taken from patients was collected into 4.9 ml tubes: K2-EDTA S-Monovette (Sarstedt, Germany). Next, peripheral blood mononuclear cells (PBMC) were isolated by density gradient centrifugation in Gradisol L (Aqua-Med, Poland) at 700 rcf for 20 minutes at room temperature. PBMC were counted in the Neubauer chamber. PBMC at a density of 1 × 10^6^ per ml were distributed into cell culture plates and stimulated at 37°C in a CO_2_ incubator for 4 hours in the presence of monensin (2 µM/ml), ionomycin (1 µg/ml) and phorbol 12-myristate 13-acetate (50 ng/ml). After stimulation, the cells were incubated with the combination of monoclonal antibodies. The following monoclonal antibodies were used in the current study: anti-TCR V alpha 24 J alpha 18 FITC (cat. no 342906, clone: 6B11, BioLegend), anti-TCR V alpha 24 J alpha 18 PE-Cy7 (cat. no 342912, clone: 6B11, BioLegend), anti-CD3 PE (cat. no 555333, BD Biosciences), anti-CD3 Krome Orange (cat. no B00068, clone: UCHT1, Beckman Coulter), anti-CD4V450 (cat. no 560345, clone: RPA-T4, BD Biosciences), anti-CD8 Alexa Fluor 700 (cat. no B76279, clone: B9.11, Beckman Coulter), anti-CD8 PE-Cy5 (cat. no 300909, clone: HIT8a, BioLegend), anti-TCR PAN delta/gamma PE-Cy5 (cat. no IM2662U, clone: IMMU510, Beckaman Coulter), anti-TCR PAN delta/gamma FITC (cat. no 347903, clone: 11F2, BD Biosciences), anti-CD45BUV395 (cat. no 563792, clone: HI30, BD Biosciences) and ViaKrome 808 (cat. no C36628, Beckman Coulter). After 20 minutes of incubation, cells were permeabilized with methanol at 4–8°C for 30 minutes in the darkness.

**Figure 3:**
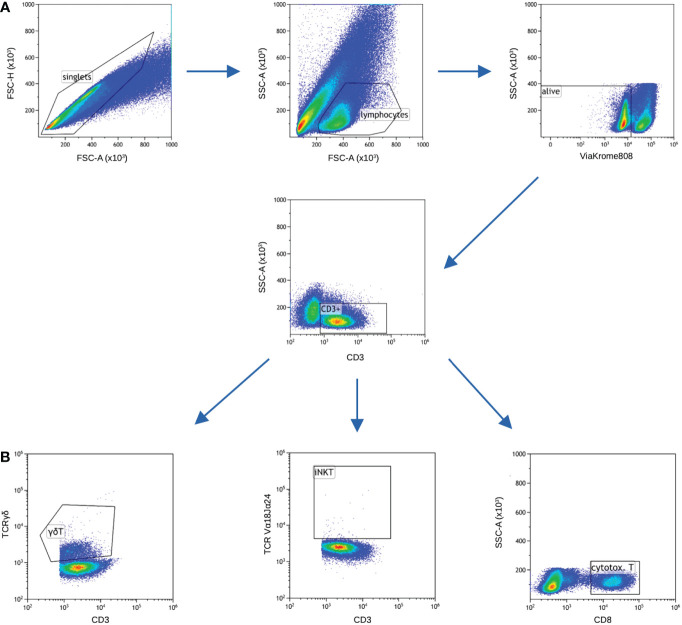
Gating strategy. Samples for cytokines and transcription factors were stained in separate tubes, nevertheless majority of gating is common for both (panels A and B), only gating of cytotoxic T cells differed. **(A)** FSC-A vs FSC-H was used to gate only single cells, next lymphocytic gate was set on FSC-A vs SSC-A. ViaKrome 808 was used to gate out dead cells. Next, CD3+ cells were gated. **(B)** Two subsets of unconventional T cells (iNKT and γδT) were gated based on specific TCR chains, cytotoxic T cells were gated as CD8+ cells among total T cells.

Next, the cells were washed twice with phosphate buffered saline (PBS) for 10 minutes, 700 rcf. Afterwards, intracellular labelling was performed using the following antibodies: anti-FoxP3 Pacific Blue (cat. no B90432, clone: 259D, Beckman Coulter), anti-IFN-gamma PE-CF594 (cat. no 562392, clone: B27, BD Biosciences), anti-IL-10 Brilliant Violet 786 (cat. no 564049, clone: JES3-9D7, BD Biosciences), anti-IL-17A APC-Cy7 (cat. no. 512320, clone: BL168, BioLegend), anti-IL-4 Brilliant Violet 605 (cat. no. 500828, clone: MP4-25D2, BioLegend), anti-E4BP4 PE (cat. no. 12-9812-42, clone: MABA223, Invitrogen), anti-T-bet APC (cat. no. 644814, clone: 4B10, BioLegend), anti-GATA-3 PerCP-eFluor 710 (cat. no. 44-9966-42, clone: TWAJ, Invitrogen) and anti-ROR gamma T Brilliant Violet 650 (cat. no. 563424, clone: RPA-T4, BD Biosciences). Incubation was performed at room temperature for 30 minutes in the darkness. After final wash with PBS, samples were analyzed on CytoFlex LX flow cytometer (Beckman Coulter, USA). Kaluza v2.1.1 (Beckman Coulter, USA) was used to analyze and graphically present the collected data (**Figure 3**).

### Sample size

Sample size was determined using sample size test in Statistica 13.3 software (StatSoft, Poland) based on previous studies on the effect of immunostimulant products on the number of CD3+ lymphocytes in children with allergic asthma, recurrent respiratory tract infections and adults with chronic obstructive pulmonary disease (40–42). It was calculated that 21 patients should be included in the PMBL and placebo groups, respectively (power 85%, alpha value 0.05). The total sample size calculation required to enroll 50 patients in anticipation of a dropout rate of 15%.

### Statistical analysis

Statistical analysis was conducted by an International Statistical Institute Elected Member not involved in the study using IBM SPSS Statistics 25 package. Using a two-way mixed model analysis of variance, checks were made to assess whether there were statistically significant differences between the study periods analyzed in the two groups, as well as whether the groups differed in terms of the variable under analysis in the same study period. Spearman’s rank correlation made it possible to check whether there was a statistically significant relationship between the analyzed variables. The Mann-Whitney U test was used to assess the presence of statistically significant differences between the two independent groups (in terms of age). A two-tailed P value lower than 0.05 was considered as statistically significant. Analyses were based on the intent-to-treat (ITT) population (patients who took ≥ 1 tablet and had ≥ 1 post-randomization assessment).

## Results

### Participant flow

**Figure 4:**
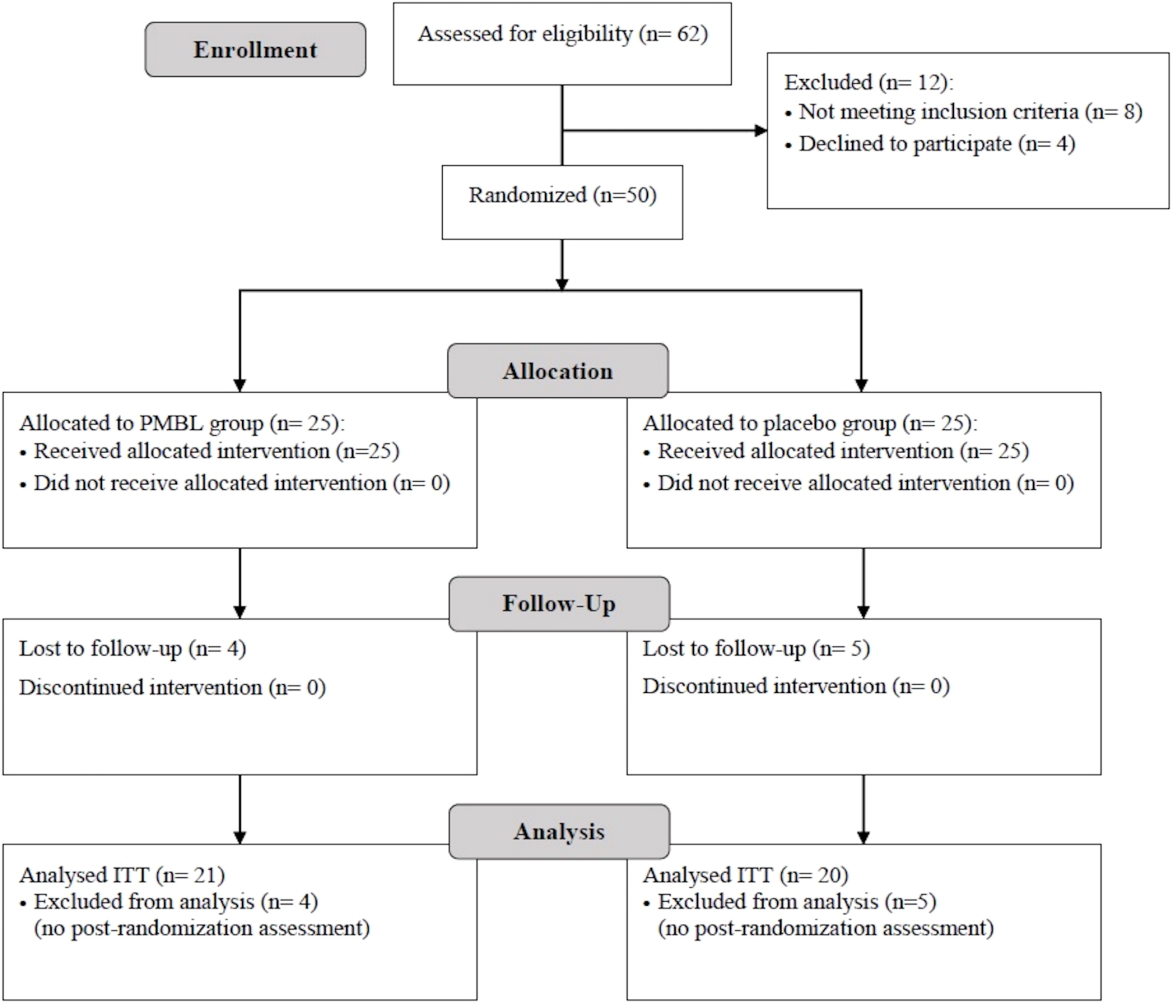
Patient flowchart (presented in accordance with the Consolidated Standards of Reporting Trials guidelines). PMBL, polyvalent mechanical bacterial lysate; ITT, intent-to-treat.

62 children were enrolled in the study. After excluding 8 patients who did not meet the inclusion criteria or met at least one exclusion criterion and 4 patients who did not agree to participate in the study, 25 children were randomized to the PMBL group and 25 to the placebo group. A total of 41 children completed the trial, 21 in the PMBL group and 20 in the placebo group (**Figure 4**). Of the 41 children, 29 (71%) were male and 12 (29%) were female. Mean age of participants was 9.1 ± 2.55 years. Demographic characteristics were similar between the groups as shown in **Table 1**.

**Table 1:** Baseline overall characteristics of patients.

	PMBL group (n = 21)	Placebo group (n = 20)	p-value
Sex, n (%)			
Male	14 (66.7)	15 (75)	0.73
Female	7 (33.3)	5 (25)	
**Age**, mean (SD) [years]	9.43 (2.68)	8.7 (2.49)	0.32
Place of residence, n (%)			
Village	7 (33.3)	6 (30)	1.00
City	14 (66.7)	14 (70)	
Sensitizing allergen, n (%)			
Grasses	21 (100)	20 (100)	–
Weeds	2 (9.5)	2 (10)	1.00
House dust mite	8 (38.1)	10 (50)	0.54
Pet dander	6 (28.6)	4 (20)	0.72
Moulds	3 (14.3)	1 (5)	0.61

PMBL, polyvalent mechanical bacterial lysate.

### Primary outcome

In both groups, during the grass pollen season, a statistically significant increase and then a decrease in the severity of SAR symptoms expressed on the TNSS and VAS was observed (**Table 2**). In the placebo group there was a statistically significant decrease in PNIF (p = 0.007), while in the PMBL group there was a statistically insignificant (p = 0.07) but clinically significant increase of 11.19 L/min (minimally clinically important difference [MCID] is 5 L/min) (**Table 3**).

**Table 2:** The severity of nasal symptoms assessed using the total nasal symptom score and visual analogue scale.

	PMBL group (n = 21)				
	V1	TC1	V2	TC2	p-value
**TNSS** ^a^, mean (SD)	0.69 (0.86)	2.22 (1.84)	1.92 (1.37)	1.12 (1.11)	V1 vs TC1 0.01 V1 vs V2 0.03 TC1 vs TC2 0.02
**VAS**, mean (SD)	9.52 (6.46)	23.24 (14.77)	22.81 (13.48)	14.27 (9.71)	V1 vs V2 0.02 V2 vs TC2 0.03
	**Placebo group** (n=20)				
	**V1**	**TC1**	**V2**	**TC2**	**p-value**
**TNSS** ^a^, mean (SD)	0.82 (0.77)	3.82 (2.15)	3.36 (1.94)	1.96 (2.19)	V1 vs TC1 < 0.001 V1 vs V2 < 0.001 TC1 vs TC2 < 0.001 V2 vs TC2 < 0.001
**VAS**, mean (SD)	7.65 (6.18)	38.25 (28.7)	34.45 (21.37)	25.35 (19.61)	V1 vs others < 0.001

PMBL, polyvalent mechanical bacterial lysate; TNSS, total nasal symptom score; VAS, visual analogue scale; V1, V2, visit 1 and 2; TC1, TC2, telephone contact 1 and 2; ^a^ TNSS symptom severity was assessed daily for 7 days after each visit, statistical analysis was performed on weekly mean values.

**Table 3:** Peak nasal inspiratory flow.

	PMBL group (n=21)		
	V1	V2	p-value
**PNIF** (l/min), mean (SD)	105.71 (40.6)	116.9 (61.57)	0.07
	**Placebo group** (n=20)		
	**V1**	**V2**	**p-value**
**PNIF** (l/min), mean (SD)	104.5 (31.11)	86.75 (28.57)	0.007

PMBL, polyvalent mechanical bacterial lysate; PNIF, peak nasal inspiratory flow; V1, V2, visit 1 and 2.

**Figure 5:**
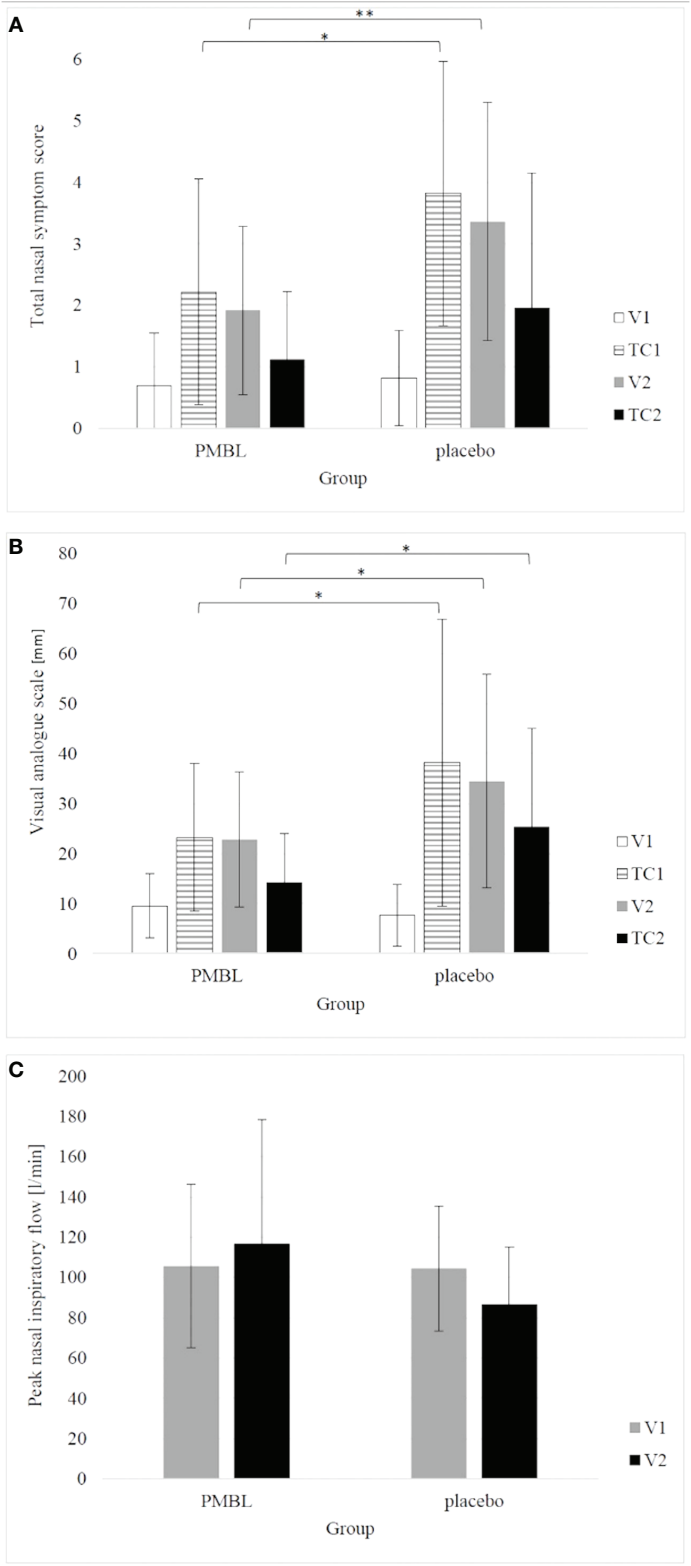
**(A)** total nasal symptom score; **(B)** visual analogue scale; **(C)** peak nasal inspiratory flow. The error bars represent standard deviation. (*p < 0.05; **p < 0.01). PMBL, polyvalent mechanical bacterial lysate; V1, V2, visit 1 and 2; TC1, TC2, telephone contact 1 and 2.

Children taking PMBL during the grass pollen season displayed much less intensity of nasal symptoms of SAR compared to children receiving placebo. The severity of the nasal symptoms of SAR on the TNSS scale was revealed to have a significantly lower impact in the PMBL group vs the placebo group at measuring points TC1 and V2 (p = 0.01, p = 0.009, respectively) (**Figure 5A**). A statistically significantly lower mean severity of nasal symptoms of SAR on the VAS scale was recorded for children in the PMBL group compared to the placebo group at measuring points TC1, V2 and TC2 (p = 0.04, p = 0.04, p = 0.03, respectively) (**Figure 5B**). The compared groups do not show significant differences in terms of PNIF values at individual measuring points, although a p-value on the verge of statistical significance (p = 0.053) was obtained at measuring point V2, indicating less nasal obstruction in the PMBL group versus the placebo group (**Figure 5C**).

**Figure 6:**
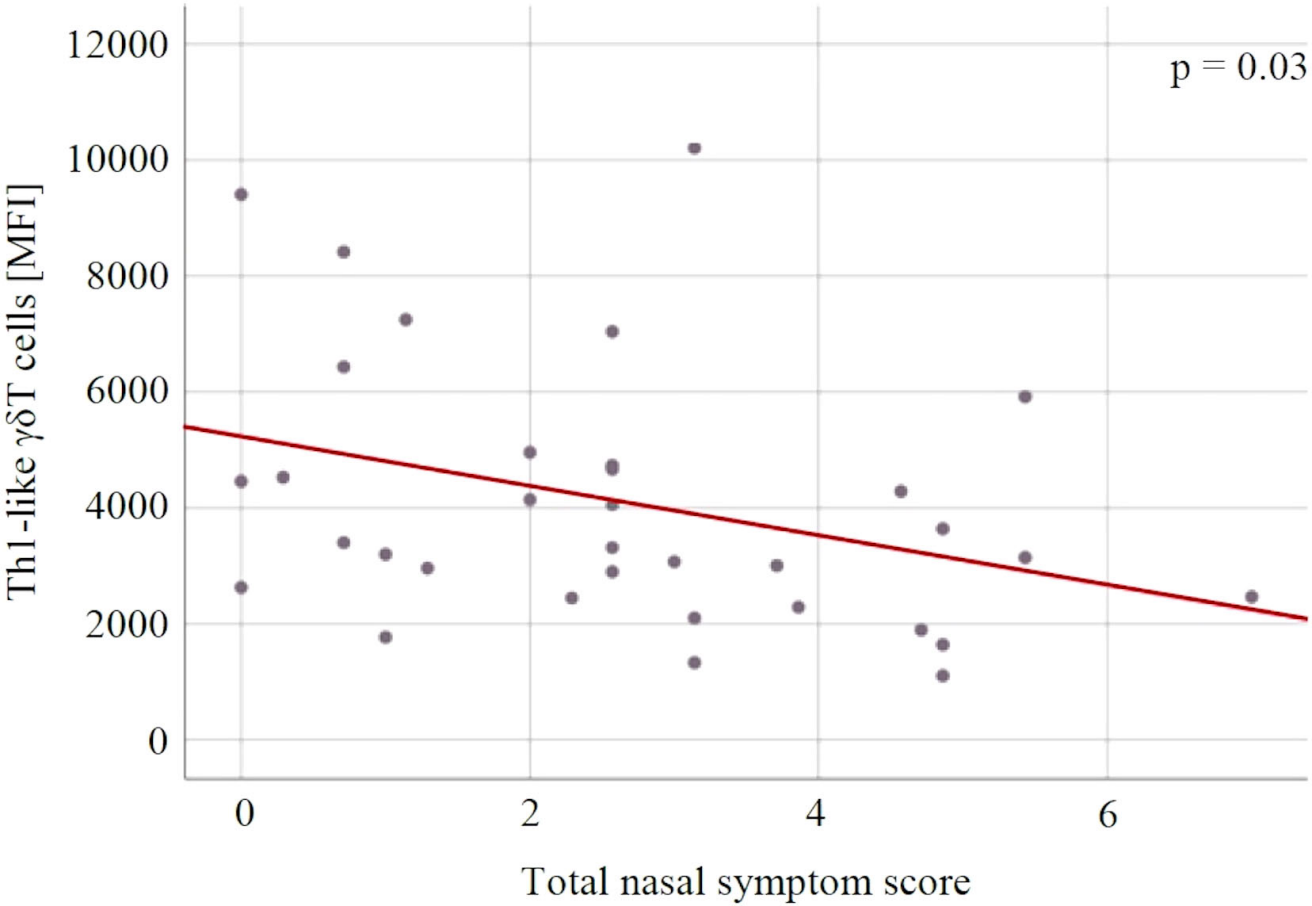
The relationship between the Th1-like γδT cells and the severity of allergic rhinitis symptoms expressed in the total nasal symptom score. MFI, mean fluorescent intensity.

There were no statistically significant changes in immune variables between V1 and V2 in both groups, and between the groups at both measurement points (**Table 4**). For both groups, there was a statistically significant association between the level of Th1-like γδT cells and the severity of SAR symptoms expressed on the TNSS scale (p = 0.03) – the lower the level of Th1-like γδT cells, the higher the TNSS value (**Figure 6**).

**Table 4:** Variation in γδT, iNKT and cytotoxic T lymphocytes subsets and cytokines throughout the study period.

Lymphocytes	Subsets/cytokines	PMBL group (n = 21)		Placebo group (n = 20)		p-value^a^
		V1	V2	V1	V2	
**γδT cells** mean (SD) [MFI]	T-bet (Th1-like)	5726.85 (3484.87)	4476.69 (2493.26)	4933.24 (4687.07)	3603.62 (1787.91)	0.63
	GATA3 (Th2-like)	4274.66 (1995.52)	4125.33 (1876.21)	3866.94 (3873.73)	3986.63 (1271.24)	0.77
	E4BP4 (Th10-like)	3392.55 (3905.4)	929.97 (220.23)	3036.41 (3256.12)	911.39 (209.6)	0.84
	RORγT (Th17-like)	340.16 (483.1)	85.75 (53.15)	285.75 (501.88)	100.15 (40.33)	0.88
	FoxP3 (Treg-like)	1786.23 (766.63)	1826.17 (816.32)	2005.91 (801.78)	5666.8 (4510.67)	0.35
	IL-4	2371.86 (835.47)	2573.19 (890.13)	2384.62 (917.63)	44336.75 (178971.66)	0.32
	IL-10	793.17 (664.88)	814.03 (706.85)	962.53 (742.4)	5211.2 (19631.84)	0.34
	IL-17A	12968.59 (11180.63)	16201.53 (13949.6)	15163.8 (13732.43)	11270.24 (10250.44)	0.28
	IFN-γ	758.8 (958.35)	623.73 (461.38)	968.38 (1122.13)	695.03 (984.39)	0.95
**iNKT cells** mean (SD) [MFI]	T-bet (iNKT1)	27441.93 (19084.15)	2737.11 (2197.81)	28015.4 (34328.33)	2610.76 (1310.58)	0.98
	GATA3 (iNKT2)	6530.14 (8944.69)	2736.17 (1391.58)	6065.95 (7690.32)	6012.15 (8977.67)	0.17
	E4BP4 (iNKT10)	3927.27 (4554.89)	1028.29 (468.21)	3776.73 (4576.69)	1124.01 (1194.18)	0.88
	RORγT (iNKT17)	3591.45 (8045.12)	119.1 (98.62)	3814.15 (8198.44)	533.74 (1512.65)	0.95
	FoxP3 (iNKTreg)	4264.5 (3056.07)	3925.12 (3436.62)	3818.86 (2835.85)	2979.88 (1335.06)	0.71
	IL-4	4994.25 (2183.97)	4663.55 (1983.26)	4720.92 (2467.46)	5407.15 (4709.77)	0.43
	IL-10	1144.93 (388.95)	1653.82 (2633.29)	2123.1 (2269.84)	1286.43 (996.84)	0.11
	IL-17A	19069.63 (16766.35)	21533.56 (20930.87)	24564.27 (20688.77)	15287.22 (11747.6)	0.16
	IFN-γ	348.34 (405.38)	732.35 (756.52)	704.08 (784.79)	568.14 (634.45)	0.08
**Cytotoxic** **T cells** mean (SD) [MFI]	T-bet (Tc1)	4325.1 (1261.6)	4658.24 (1594.22)	3977.56 (1026.32)	4217.21 (1170.27)	0.43
	GATA3 (Tc2)	3798.56 (2132.26)	2012.49 (2183.65)	3515.95 (3526.99)	5743.84 (1321.61)	0.89
	E4BP4 (Tc10)	3899.59 (4869.09)	666.39 (508.88)	3648.8 (4409.43)	610.52 (438.2)	0.69
	RORγT (Tc17)	288.66 (236.78)	37.34 (31.81)	221.4 (359.53)	40.05 (33.07)	0.9
	FoxP3 (Treg-like)	1726.32 (733.62)	1522.49 (463.29)	1848.78 (704.86)	1630.25 (609.61)	0.88
	IL-4	2298.71 (835.18)	2223.63 (679.1)	2162.73 (873.79)	2068.96 (609.24)	0.9
	IL-10	931.94 (752.56)	703.49 (557.84)	975.45 (817.07)	789.32 (773.14)	0.53
	IL-17A	12602.21 (10871.44)	12626.69 (9680.75)	13749 (11957.41)	13456.06 (10917.35)	0.87
	IFN-γ	832.33 (743.81)	747.11 (619.32)	947.52 (1093.35)	530.92 (392.53)	0.63

PMBL, polyvalent mechanical bacterial lysate; V1, V2, visit 1 and 2; MFI, mean fluorescent intensity.

a The analysis of variance showed no statistically significant interaction of the group with the level of measurement for individual variables, meaning that the two groups do not differ in V1 and V2 for individual variables, and that there are no differences between V1 and V2 in individual groups.

Data is presented as mean fluorescent intensity (MFI).

### Secondary outcome

The mean number of days of use of desloratadine and mometasone furoate per patient was respectively 26% (18.62 ± 4.6 vs 25.2 ± 7.4 days, p = 0.005) and 31% (28.57 ± 7.41 vs 41.6 ± 11.17 days, p < 0.001) lower in the PMBL group vs the placebo group. The therapy was associated with a high safety and tolerability profile. In the PMBL group only one adverse event in forms of mild abdominal pain was reported, which resolved spontaneously without any medication. No adverse event was reported in the placebo group.

## Discussion

As mentioned earlier, the present study was designed to evaluate the efficacy of PMBL therapy in children with SAR, and primarily to further understand the immune mechanisms of bacterial immunostimulants responsible for reducing the severity of allergic disease symptoms. To the best of our knowledge, this is the first study to evaluate the effect of PMBL therapy on changes of γδT, iNKT and Tc cell subsets in the serum levels in children with AR.

The study presented here is a continuation of a series of studies on the efficacy of BLs in the treatment of grass pollen-induced AR in children, but it focuses primarily on the immunological mechanisms of these preparations. The results of the first project were published in 2019. It was a pilot, randomized, open-label study that included 38 children with SAR. The addition of sublingual BL to standard AR treatment (oral antihistamine, intranasal corticosteroid) has been shown to reduce the severity of nasal symptoms as assessed by a standard scoring scale and to improve nasal patency expressed as an increase in PNIF values. Nevertheless, the drug has not been confirmed to alleviate symptoms of allergic conjunctivitis (43). As a clinically significant reduction in nasal symptom severity on the TNSS scale was found (a reduction of 1.54, with a MCID of 0.55), decision was made to conduct a randomized, double-blind, placebo-controlled trial in the next grass pollen season. The project involved 70 children who were randomly assigned to a group receiving PMBL or placebo. During the grass pollen season, an improvement in the clinical course of SAR was observed, expressed as a statistically significant decrease in TNSS (p = 0.001) and VAS values (p < 0.001) and a statistically significant increase in PNIF values (p = 0.04) in the PMBL treatment group. Patients taking the immunostimulant had significantly lower nasal symptom severity than children in the placebo group. The compared groups did not differ in the severity of ocular allergy symptoms. In addition, the use of PMBL was associated with a reduction in the need for desloratadine and mometasone furoate. In order to assess the effect of PMBL therapy on Th1/Th2 balance, nasal swabs were taken from the patients for eosinophils and nasal lavage fluids for asIgE against timothy grass. Relation between the number of eosinophils in nasal swabs and the concentration of grass pollen grains in ambient air was recorded for both groups, although the number of these cells was significantly lower in the PMBL group (p = 0.01). Furthermore, no changes in asIgE concentrations during the grass pollen season was recorded in the PMBL group, while in the control group, these concentrations increased significantly (p = 0.03). The above observations indirectly indicate the influence of PMBL on the suppression of the dominant Th2 lymphocyte response in SAR patients (20). Similar conclusions can be drawn based on the results of the present study, which showed statistically significant lower severity of SAR symptoms expressed on the TNSS and VAS scales, as well as lower allergy medication use in the PMBL treatment group vs the placebo group. A clinically significant increase in PNIF values during the grass pollen season was observed in the PMBL group, while a significant decrease was observed in the placebo group, and the differences found between groups on this variable were close to the level of statistical significance. The beneficial effect of BL therapy on the clinical course of AR in adult patients was also confirmed by Banche et al. (21) and Meng et al. (23). Italian researchers have shown that PMBL therapy reduces the severity of AR symptoms (in 62% of patients) and improves asthma control (in 40% of patients), with the achieved effects lasting at least 3 months. In contrast, in the placebo group, more than half of the patients experienced a worsening of AR symptoms. The authors signal that the achieved effects of PMBL therapy may be related to a significant reduction in IL-4 levels in the blood (21). On the other hand, Meng et al. evaluated the efficacy of PCBL in adult patients with PAR, demonstrating, like us, the effect of immunostimulation with the bacterial product on reducing nasal symptoms and reducing the need for oral antihistamines by almost 40%. Moreover, the researchers demonstrated the effect of these preparations on reducing Th2-type cytokines (IL-4 and IL-13) and increasing Th1-type cytokine (IFN- γ) (23). The role of BLs in restoring and maintaining Th1/Th2 balance, as well as other mechanisms of action of BLs in allergic diseases known so far, have been thoroughly discussed by us in other articles (44, 45).

Recent data indicate that Tc cells play a role in the chronic inflammatory diseases, including allergic ones (46). Exposure to specific antigens can trigger these cells to release inflammatory molecules. In AR, researchers have noted that Tc cells release IL-4, which is involved in the pathogenesis of the disease, and contributes to the production of asIgE by B lymphocytes (47). γδT and iNKT cells are also able to produce and release large amounts of pro- and anti-inflammatory cytokines and chemokines after being stimulated by several factors, such as microbial antigens (27, 32). Rapid synthesis of cytokines makes these cells important regulators of inflammatory processes, including allergic processes. It is postulated that γδT and iNKT cells are involved in the development and modulation of the course of allergic diseases (30, 31, 34).

It has been shown that some molecules can modulate the activation of the above-mentioned cells. Johansson et al. investigated if lactobacilli-derived factors could beneficially change immune responsiveness of γδT and NK cells *in vitro*. They showed that molecules present in the lactobacilli cell-free supernatants act directly on these cells, reducing their activation, which provided a novel insight on the immunomodulatory nature of probiotic lactobacilli (48). Wang et al. showed that probiotic treatment (mixture of lactobacillus, bifidobacterium, and Streptococcus thermophilus*)* can restore adipose iNKT cell frequency and enhance the function of the iNKT cell anti-inflammatory phenotype in high-fat diet-induced obese mice (49). Furthermore, Olszak et al. concluded that early-life colonization of germ-free mice with a conventional microbiota protected them from mucosal iNKT accumulation and related pathologies such as asthma and inflammatory bowel disease (50). Mentioned studies suggest that some bacteria molecules can influence the activation of γδT and iNKT cells. It can be presumed that BLs, as preparations consisting of inactivated antigens derived from respiratory pathogens, are also able to modulate the immune response by restoring these cells balance. However, our study did not confirm this hypothesis. To the best of our knowledge, our study is the first to evaluate the effect of BL therapy on changes of γδT, iNKT and Tc cell subsets in the serum levels in children with AR. Our lack of confirmation of the results of previous studies may be due to the fact that they involved probiotics, were *in vitro* or animal studies, and were not conducted on an allergic disease model. It is worth mentioning that we focused mostly on unconventional T cells that undergo significant changes during childhood and early adolescence. This is especially true for γδT cells. At birth the Vδ1 subset prevails, it comprises usually more than 95% of total circulating γδT cells. In adults Vδ2 dominates, while Vδ1 rarely exceeds 25% of total γδT in peripheral blood. Both subsets recognize different stimuli and may respond in slightly different ways (28). Thus, data obtained in children and adults is usually not fully comparable.

γδT can be divided similarly to the conventional T helper cells into populations based on their cytokine profile and transcription factors. At least the major subsets (Th1-, Th2-, Th10-, Th17- and Treg-like) can be distinguished (51). γδT percentage in peripheral blood tends to be significantly lowered in asthmatic patients, which suggests a possible role in asthma and allergy pathogenesis (28). Moreover, γδT may be also involved in regulation of IgE production, this observation has been so far noted only in murine model of asthma, not in patients’ samples (52). In the current study, we have observed a decrease in Th1-like (T-bet+) γδT cells with the increase in allergic symptoms expressed on the TNSS scale. This is generally in line with the general theory of immunopathogenesis, namely the dysregulation of Th1/Th2 balance.

Our study has some limitations. First, a longer follow up, more numerous population and more detailed studies are needed to elucidate the mechanism of action of PMBL in patients with AR. Second, since we have only studied systemic effects, it is possible that different or stronger immune effects can be found locally in the respiratory tract.

## Conclusion

Administration of sublingual PMBL tablets during the grass pollen season proves to have a high efficacy in alleviating SAR symptoms in children sensitized to grass pollen allergens. PMBL treatment has not been shown to alter the levels of individual subsets of γδT, iNKT and Tc cells. Th1-like γδT cells may be used as potential markers for SAR severity in children.

## Data availability statement

The raw data supporting the conclusions of this article will be made available by the authors, without undue reservation.

## Ethics statement

The studies involving human participants were reviewed and approved by Bioethics Committee of the Medical University of Lublin. Written informed consent to participate in this study was provided by the participants’ legal guardian/next of kin.

## Author contributions

Conceptualization: KJ and MZ; methodology: KJ, WK, MZ, AE, and JR; investigation: KJ, WK, MZ, DS, MM, EM-M, IM-M, AB, AT, and KP-W; data interpretation: KJ, WK, and MZ; supervision: KJ, AE, and JR; writing original draft: KJ, WK, MZ, DS, IM-M, AB, AT, AK, AE, and KP-W. All authors contributed to the article and approved the submitted version.
